# Acute and Chronic Effects of Particles on Hospital Admissions in New-England

**DOI:** 10.1371/journal.pone.0034664

**Published:** 2012-04-17

**Authors:** Itai Kloog, Brent A. Coull, Antonella Zanobetti, Petros Koutrakis, Joel D. Schwartz

**Affiliations:** 1 Exposure, Epidemiology and Risk Program, Department of Environmental Health, Harvard School of Public Health, Boston, Massachusetts, United States of America; 2 Department of Biostatistics, Harvard School of Public Health, Boston, Massachusetts, United States of America; University of Swansea, United Kingdom

## Abstract

**Background:**

Many studies have reported significant associations between exposure to PM_2.5_ and hospital admissions, but all have focused on the effects of short-term exposure. In addition all these studies have relied on a limited number of PM_2.5_ monitors in their study regions, which introduces exposure error, and excludes rural and suburban populations from locations in which monitors are not available, reducing generalizability and potentially creating selection bias.

**Methods:**

Using our novel prediction models for exposure combining land use regression with physical measurements (satellite aerosol optical depth) we investigated both the long and short term effects of PM_2.5_ exposures on hospital admissions across New-England for all residents aged 65 and older. We performed separate Poisson regression analysis for each admission type: all respiratory, cardiovascular disease (CVD), stroke and diabetes. Daily admission counts in each zip code were regressed against long and short-term PM_2.5_ exposure, temperature, socio-economic data and a spline of time to control for seasonal trends in baseline risk.

**Results:**

We observed associations between both short-term and long-term exposure to PM_2.5_ and hospitalization for all of the outcomes examined. In example, for respiratory diseases, for every10-µg/m^3^ increase in *short-term* PM_2.5_ exposure there is a 0.70 percent increase in admissions (CI = 0.35 to 0.52) while concurrently for every10-µg/m^3^ increase in *long-term* PM_2.5_ exposure there is a 4.22 percent increase in admissions (CI = 1.06 to 4.75).

**Conclusions:**

As with mortality studies, chronic exposure to particles is associated with substantially larger increases in hospital admissions than acute exposure and both can be detected simultaneously using our exposure models.

## Introduction

Short-term variations in air pollution have been associated with hospital admissions for various causes in cities all over the world [1], [2], [3], [4], [5], [6]. These associations include admissions for respiratory disease [7], [8], [9], ischemic heart disease-IHD [10], [11], cardiovascular disease-CVD [7], [12], myocardial infarction-MI [13], [14], congestive heart failure-CHF [15], [16], pneumonia [17], [18], and diabetes [19], [20].

For PM_2.5_ in particular Dominici and colleagues [21] reported associations with hospitalizations for multiple diseases, using single day average PM_2.5_. Zanobetti and colleagues [22] estimated the association between two-day mean PM_2.5_ and emergency hospital admissions for CVD,MI,CHF, respiratory disease, and diabetes in 26 US communities, and reported larger effect sizes than those reported in Dominici et al. [21]. There are currently, to the best of our knowledge, no published studies on the effects of long-term (chronic) particulate matter (PM) exposure and hospital admissions. There are however some studies that provide general evidence for long-term associations of air pollution with hospital admissions, although not specifically focusing on PM_2.5_ [23], [24], [25], [26]. For example, Oudin and colleagues [25] investigated whether the effects of major risk factors for ischemic stroke were modified by long-term exposure to air pollution in Scania, southern Sweden. They found that in low level air pollution areas, the risk for ischemic stroke associated with diabetes seemed to increase with long-term exposure to air pollution. Hruba and colleuges [24] studied the effects of long-term exposure to air pollution on respiratory symptoms and respiratory hospitalization in a cross-sectional study of children. They showed found a significant increase in hospital admissions for asthma, bronchitis or pneumonia associated with increasing air pollution. Andersen and colleagues [26] studied the association between chronic exposure to traffic-related air pollution (NO_2_) and incidence of diabetes. They found that chronic exposure to NO_2_ may contribute to the development of diabetes, especially in individuals with a healthy lifestyle, nonsmokers, and physically active individuals.

All previous studies have been limited by the lack of high resolution daily exposure data. Many early studies had only 1 in 3 day measurements, and locations without nearby monitors could not be analyzed at all. In addition all previous studies focused on short-term PM exposure and not long term (chronic) exposure or both.

We have recently presented a new method of assessing temporally-and spatially-resolved PM_2.5_ exposures for epidemiological studies which is an extension of existing land use models [27], [28]. In this paper, we use our model predictions to study the association between PM_2.5_ exposure and hospital admissions among elderly (aged 65 and older from Medicare data) across New England, and to investigate the effects of both short term (acute) and long-term (chronic) exposure on these outcomes for the first time concurrently. In addition our study investigates the entire population of a region, rather than selected locations near monitoring sites as commonly done in previous studies.

## Methods

### Study domain

**Figure 1:**
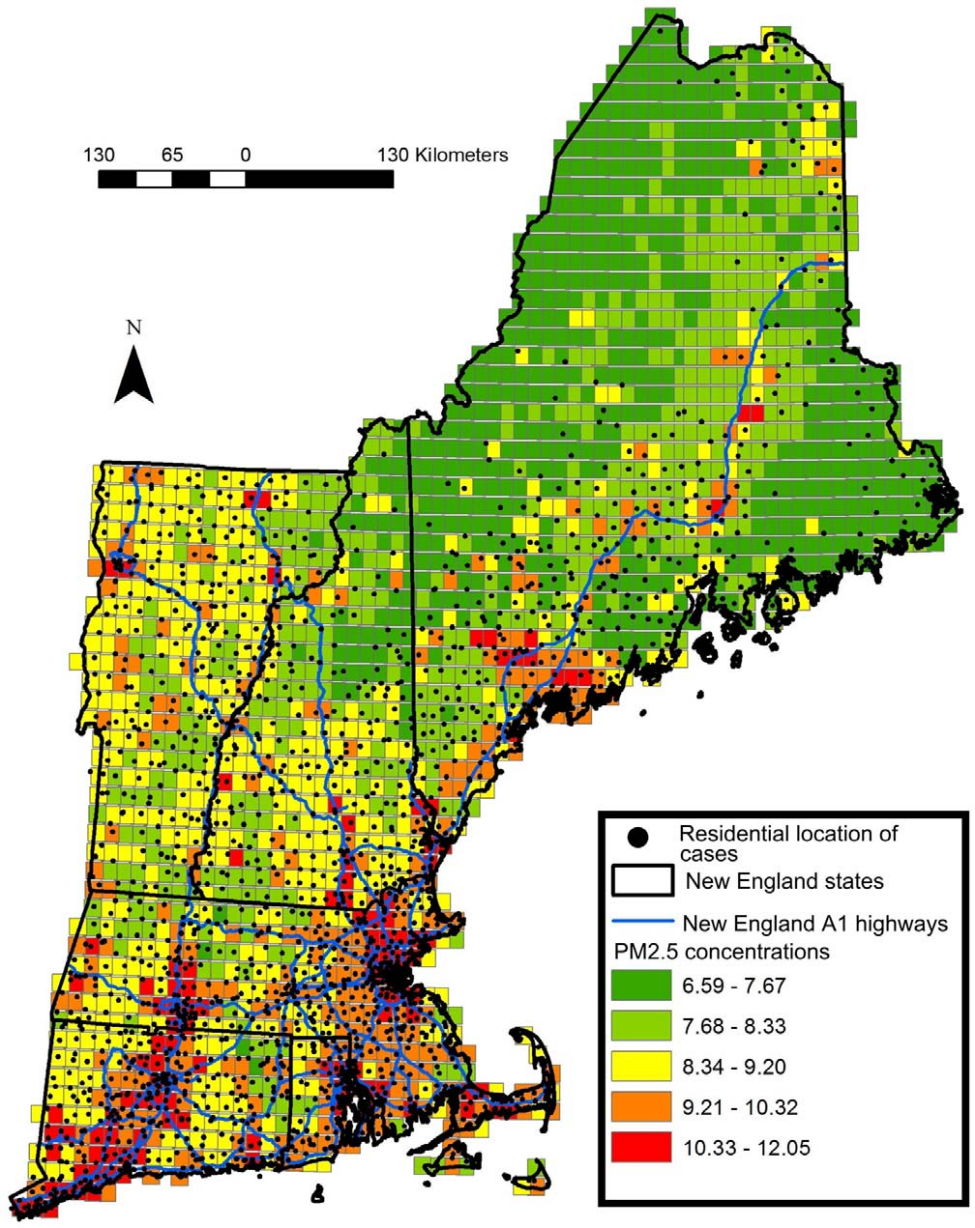
Map of the study area showing the residential location of admission cases juxtaposed over a sample PM2.5 10×10 km pollution grid for 01/07/2001.

The presented study's spatial domain included the New-England region comprising the states of Connecticut, Maine, Massachusetts, New Hampshire, Rhode Island and Vermont, (Figure 1). The total area of New England is 186,460 km^2^. The total population in New-England as of 2010 is 14,444,865. The average size of population in New-England zip codes for the general population is 8130 and 1105 for people 65 and over. The median population is 3535 for the general population and 430 for people 65 and over [29].

### Data

#### Exposure data

Land use regression (LUR) models provide good estimates of spatially resolved long term exposures, but are poor at capturing short term exposures. Due to its large spatial coverage and reliable repeated measurements, satellite remote sensing, provides another important tool for monitoring aerosols, particularly for areas and exposure scenarios where surface PM_2.5_ monitors are not available [30], [31], [32], [33]. Using satellite derived aerosol optical depth (AOD) measurements allowed us to predict daily PM_2.5_ concentration levels across New England for 2000–2008 at a 10×10 km spatial resolution [34]. This published model has been slightly updated to include nested regions in the yearly models and weights to account for non-random missingness in AOD.

In brief, we used day-specific calibrations of AOD data, using ground PM_2.5_ measurements from 78 monitoring sites in the EPA (Environmental Protection Agency) and IMPROVE (Interagency Monitoring of Protected Visual Environments) monitoring network to avoid prediction error due to changes in planetary boundary layer etc. previously noted by Paciorek et al. [28]. We also incorporated land use regression and meteorological variables (temperature, wind Speed, visibility, elevation, distance to major roads, percent of open space, point emissions and area emissions). To estimate PM_2.5_ concentrations in each grid cell on each day we start by calibrating the AOD-PM_2.5_ relationship for each day using grid cells with both monitors and AOD values using mixed models with random slopes for day and nested regions. To validate our first model, the dataset was repeatedly randomly divided into 90% and 10% splits. Predictions for the held-out 10% of the data were made from the model fit of the remaining 90% of the data. This “out of sample” process was repeated ten times and cross-validated (CV) R^2^ values were computed. The first stage calibrations resulted in high out-of-sample R^2^ (mean out-of-sample R^2^ = 0.85). Later, we used a second model to address days when *AOD measures are not available* (due to cloud coverage, snow etc...). We thus fit a model with a smooth function of latitude and longitude and a random intercept for each cell (similar to universal kriging) that takes advantage of the association of grid cells AOD values with PM_2.5_ monitoring located elsewhere, and the association with available AOD values in neighboring grid cells. Even for location-day combinations without AOD data our model performance was still excellent (mean out-of-sample R^2^ = 0.81). Importantly, these R^2^ are for daily observations, rather than monthly or yearly, values. By averaging our estimated daily exposures at each location we generated long term exposures. This enabled us to study both the short term and long term effects of ambient particles, respectively.

PM_2.5_ exposure data were generated by our prediction models. The New-England exposure dataset contains daily PM_2.5_ concentrations at a 10×10 km spatial resolution across New-England for the whole study period (Figure 1). This data was matched to zipcodes using ArcGIS and SAS based on spatial location and date.

#### Hospital Admittance data

Individual hospital admittance records were obtained from the US Medicare program and covers hospitalization for all residents aged 65 and older, for all available years (2000–2006). There were around 3000 hospitals under the study area. We defined cases as those with an emergency admission and a primary discharge diagnosis of all respiratory (ICD 9 460–519), CVD (ICD 9 390–429), stroke (ICD 9 430–436) and diabetes (both primary and secondary admission cause) (ICD 9 250).

We choose broader areas of admissions, since one would expect broader areas of admission to produce less noisy estimates for two reasons. First, the counts are higher and therefore there is more power to examine CVD admissions than IHD admissions. Secondly, studies of misdiagnosis in hospital administrative records show that the broader the categories, the less misclassification there is, which would also eliminate noise and produce more stable results. For diabetes, which is a chronic condition, we looked at the rate of admission of subjects for any primary cause with diabetes as a secondary cause, as well as the small number of admissions with diabetes listed as the primary cause of admission. This allows us to examine whether long term exposure to particles is associated with higher rates of hospitalization of diabetics, as well as whether diabetics have higher rates of acute hospitalizations on high air pollution days.

These records included information such as age, sex, date of admission, race/ethnicity, and zipcode of residence. From this data, we constructed daily counts for each admission cause for each zip code. This allows us to examine the effects of both day-to-day contrasts within residential area, as well as long term contrasts across locations.

#### Covariates

Temperature data were obtained through the National Climatic Data Center (NCDC) [35]. Only continuous operating stations with daily data running from 2000–2006 were used. Zipcodes were matched to the closest weather station for meteorological variables. All *Socioeconomic* variables were obtained through the U.S. Census Bureau Census from the 2000 social, economic and housing characteristics datasets [36]. S*ocio-economic* variables used included the following zipcode level information: Percent of minorities, age, education (people with no high school education) and median income.

### Statistical Methods

The admission counts by zip code were matched with our exposure estimates for each 10×10 km grid cell it fell into. While short-term effects of air pollution are traditionally studied using Poisson log-linear models and long-term effects are estimated using the Cox proportional hazard model, we make use of the equivalence between Poisson regression and the piecewise constant proportional hazard model noted by Laird and Oliver [37]. This approach allows us to model the time to a hospital admission as a function of both long term and short term exposure simultaneously. Most time series studies have reported stronger associations with mean PM_2.5_ taken over the current and previous day as compared to same day exposure [38]; therefore for the short-term exposure we used the mean of PM_2.5_ on the day of admission and day before admission in all models. Long-term exposure was calculated as the mean exposure in each zip-code across the whole study period (7 years). Short term exposure was defined as the difference between the two day average and the long-term average. To check the linearity of main effects investigated we fit a piecewise linear model estimating the effect of PM for levels below and above the median of short and long term PM_2.5_. We did not find a significantly different effect between the two slopes above and below the median which suggests a linear relationship of these variables.

**Table 1:** Descriptive statistics stratified by long term exposure: Hospital admissions by type of admission across New-England for the years 2000–2006.

Characteristic	All Respiratory	CVD	Stroke	Diabetes
	No. (%)	No. (%)	No. (%)	No. (%)
**Low pollution**				
**Sex**				
Male	89241 (44.63)	131234(45.52)	24066(41.71)	77553(43.59)
Female	11073 (55.37)	157039(54.48)	33638(58.29)	100382(56.41)
**Race**				
White	192257 (94.41)	277404(96.23)	55112(95.51)	165174(92.83)
Black	3321 (1.66)	4885(1.69)	1186(2.06)	6339(3.56)
other	4395 (2.20)	5984(2.08)	1406(2.44)	6422(3.61)
Age	79.55	79.24	80.30	77.24
**High pollution**				
**Sex**				
Male	101629(44.52)	148566(44.55)	27516(40.66)	93918(42.56)
Female	126658 (55.48)	184948(55.45)	40162(59.34)	126743(57.44)
**Race**				
White	213519 (93.53)	312202(93.61)	62741(92.71)	194360(88.08)
Black	7672 (3.36)	11920(3.57)	2920(4.31)	15682(7.11)
other	7096 (3.11)	9392( 2.82)	2017(2.98)	10619(4.81)
Age	79.64	79.31	80.27	77.26

The basic model takes advantage of the fact that a hierarchical mixed Poisson regression can capture both acute and chronic effects. Specifically, we assume that the admission rate λ_it_ in the i^th^ cell on the t^th^ day can be modeled as follows:
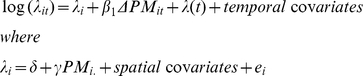
where λ_i_ is the long term admission rate in grid cell i, ΔPM_it_ is the deviation of the PM_2.5_ concentration in cell i from its long term average on day t, λ(t) is a smooth function of time, temporal covariates are temperature and day of the week, PM_i._ is the long term average PM concentration in cell i, spatial covariates are socioeconomic factors defined at the zipcode level, and e_i_ is the remaining unexplained difference in admission rate between cell i and other cells, which is treated as a mean zero normal random effect with variance estimated from the data. This model combines the usual Poisson time series analysis with a Poisson representation of a piecewise-constant proportional hazards model. The resulting model specifies that each time interval defining the constant hazards has a separate intercept, and an offset representing person-time at risk. Since the entire population is being analyzed, and specific admissions cases by type are rare events, the person-time at risk varies slowly and smoothly across time. In the limit as the time interval gets small, the time-period specific intercept also approaches a smooth function of time, and hence both can be replaced with the smooth function of time, λ(t).

**Table 2:** Descriptive statistics for short term PM_2.5_ exposure, long term PM_2.5_ exposure and temperature in New-England for 2000–2006.

Covariate	Mean	Min	Max	Median	SD	Range	IQR	Q1	Q3	Days of data available
**Lag0 PM_2.5_ (acute PM)**	9.60	0.01	72.59	8.55	4.90	72.59	5.32	6.35	11.67	2557
**1 year PM_2.5_ (Chronic PM)**	9.65	3.54	17.79	9.65	0.81	14.25	0.98	9.16	10.14	2557
**Temperature**	46.52	−23.80	90.10	47.90	18.73	113.90	29.30	33.00	62.30	2557

Note: Q1 and Q3 are quartiles.

The specific covariates we used were temperature with the same moving average as PM_2.5_, age, percent minorities, median income and percent of people with no high school education. λ(t) was estimated with a natural cubic spline with 35 degrees of freedom (5 df per year).

To investigate the robustness of our results various sensitivity analysis were run on the all respiratory admission as a sample group. We analyzed other averaging periods (lag1,lag2 vs lag0) and the addition of the land use and temporal variables (percent of, percent of house owners living in owned house, Percent of occupied housing units with more than one person per room and median home value, absolute humidity).

We also wanted to compare the results from our novel prediction models with an analysis of this data base using a traditional time series approach. We ran the analysis for the Boston area (Suffolk, Norfolk and Middlesex counties) using daily admission counts for all respiratory admissions, PM_2.5_ from a central PM monitor (Countway monitor at Harvard school of public health) and temperature data from Logan airport as commonly done in time series analysis.

## Results

Descriptive statistics stratified by long term pollution (high and low split by the mean) are presented in Table 1. The majority of people included in our analyses which were admitted to hospital were white (84.92%–96.23% across all admission causes) while the average age was 76.65–80.30 years.

**Table 3:** Estimated percent increase in hospital admissions for a 10 μg/m^3^ increase for both short term and long term PM_2.5_ by cause of admission.

PM_2.5_ exposure type	All Respiratory	CVD	Stroke	Diabetes
	Percent increase ^a^	Percent increase ^a^	Percent increase ^a^	Percent increase ^a^
**Short term PM_2.5_ exposure**	0.70 (0.35–0.52)	1.03 (0.69–0.45)	0.24(−0.13–0.56)	0.96(0.62– 0.51)
**Long term PM_2.5_ exposure**	4.22(1.06–4.75)	3.12(0.30–4.29)	3.49 (0.09–5.18)	6.33(3.22– 4.59)

Note: ^a^Values are percent.

Table 2 contains a summary of the predicted exposures for both the acute exposure (0 day lag) and the chronic exposure (365 day moving average) across all grid cells in the analysis.

**Table 4:** Sensitivity analysis (estimated percent increase in hospital admissions for a 10 μg/m^3^ increase in chronic PM_2.5_ exposure) for all respiratory admission causes.

PM_2.5_ exposure type	All Respiratory-acute PM_2.5_	All Respiratory-chronic PM_2.5_
	Percent increase ^a^	Percent increase ^a^
**Baseline**	0.70(0.35–0.52)	4.22(1.06–4.75)
**Added SES variable**	0.70(0.35–0.52)	3.84(0.67–4.74)
**Added temporal variable lag 0**	0.58(0.23–0.52)	4.40(1.36–4.56)
**Added temporal variable lag 01**	0.35(0.01–0.52)	4.65(1.60–4.57)
**Added temporal variables lag 02**	−0.18(−0.52 – −0.52)	5.32(2.25–4.57)

Note: ^a^Values are percent.

Table 3 presents the estimated percent increase in hospital admissions for a 10 µg/m^3^ increase for both short term and long term PM_2.5_ by cause of admission and associated 95% confidence intervals. For all respiratory, for every 10-µg/m^3^ increase in short term PM_2.5_ exposure there is a 0.70 percent increase in admissions (95% CI = 0.35 to 0.52) while concurrently for every 10-µg/m^3^ increase in long term PM_2.5_ exposure there is a 4.22 percent increase in admission (95% CI = 1.06 to 4.75). For CVD, for every10-µg/m^3^ increase in short term PM_2.5_ exposure there is a 1.03 percent increase in admission rate (95% CI = 0.69 to 0.45) while concurrently for every10-µg/m^3^ increase in long term PM_2.5_ exposure there is a 3.12 percent increase in admission (95% CI = 0.30 to 4.29). For strokes, for every 10-µg/m^3^ increase in short term PM_2.5_ exposure there is a 0.24 percent increase in admissions (95% CI = −0.13 to 0.56) while concurrently for every10-µg/m^3^ increase in long term PM_2.5_ exposure there is a 3.49 percent increase in admissions (95% CI = 0.09 to 5.18). Finally for diabetes, for every10-µg/m^3^ increase in short term PM_2.5_ exposure there is a 0.96 percent increase in admissions (95% CI = 0.62 to 0.51) while concurrently for every10-µg/m^3^ increase in long term PM_2.5_ exposure there is a 6.33 percent increase in admissions (CI = 3.22 to 4.59).

The results from the sensitivity analysis are presented in table 4. In general the results of the sensitivity analysis were consistent with the primary analysis for the added spatial variables and added temporal variable as well as for the different lags (excluding the acute PM_2.5_ exposure in lag02).

The results from the classic times series analysis were similar to the main model (1.51 vs 0.72 percent change) albeit with higher standard error (0.002 vs 0.001) and much larger CI (0.42–1.65 Vs. 0.35–0.52).

The crude and final estimates as well as the estimates for the model covariates are presented in appendix S1 and S2.

## Discussion

In this paper we report, for the first time, that long term exposure to PM_2.5_ is associated with increased hospital admissions of the elderly (aged 65 and older) for all respiratory, CVD, stroke, and diabetes. As with mortality studies, this long term impact is higher than the acute effects. Importantly, we continue to see acute effects independent of the chronic effects. In addition, this analysis covers all zip codes in New England, not just subset zipcodes locations near PM_2.5_ monitors. This represents an important extension of previous Medicare analyses, since we now have estimates that include suburban, small town, and rural populations. Finally, the use of a spatiotemporal model reduces exposure misclassification that exists in, for example time series studies that use a single exposure metric for daily exposure in an entire metropolitan area. Such error is a mixture of classical exposure error, which likely biases the effect estimates downward, and Berkson error, which increases the confidence interval [39]. The results from our novel method presented much tighter confidence intervals compared to the classic time series analysis, indicating that our method could potentially reduce measurement error. Another advantage our method adds is the ability to include population that lives far from monitor compared to the traditional methods

One of the key components of this study is that we showed that by using our prediction models (which produce daily PM_2.5_ predictions) we are able to simultaneously examine short term and long term association with hospital admissions and to do it for the entire population of New England, avoiding issues of selection or non-representative samples, and accounting for small area measures of potential confounders.

The putative biological mechanisms linking both short term and long term exposure to air pollution and CVD involve direct effects of pollutants on the cardiovascular system, blood, and lung receptors, and/or indirect effects mediated through pulmonary oxidative stress and inflammatory responses [40]. The biological mechanisms linking both short term and long term exposure to air pollution and respiratory diseases include reduced lung function, pulmonary inflammation and oxidative stress [41]. Further, an intervention trial of air filtration for elderly adults reduced particles levels and reported improved endothelial function [42]. Similarly, a trial comparing blood pressure when subjects were walking in Beijing with our without a particle filter reported blood pressure was lower when wearing the filter208.

These studies are also supported by toxicologic indicators of mechanism. For example, a recent study of mice genetically prone to atherosclerosis and on a high fat western diet exposed to concentrated particles from the outside air showed that the particle exposure lead to more atherosclerotic plaque, and increased macrophages and tissue factor in the plaques, which reduce plaque stability and increase the risk of a heart attack [43]. Another study, using a different mouse model of atherosclerosis, documented that particle exposure increased oxidation of LDL, increased the thickness of the arterial wall, and promoted plaque growth and instability [44]. A number of studies have directly linked particle exposure with ischemia. Wellenius exposed dogs to either filtered air or concentrated air particles, followed by a temporary occlusion of the coronary artery. The animals exposed to particles experienced greater ischemia than those exposed to filtered air [45], [46].

Several studies suggested an enhanced susceptibility of people with diabetes to exposure to air pollution partly due to inflammatory mechanisms [19], [20], [47]. In addition there are reports associating air pollution with incidence of diabetes [48].

Our estimated associations between short term exposure to PM_2.5_ and hospital admissions revealed results qualitatively similar to those studies previously published analyzing short term PM_2.5_ and hospital admissions [10], [21], [49]. To the best of our knowledge there are no studies on exposure to long term PM_2.5_ and hospital admissions. However our long term exposure results for CVD are in agreement with those reported by Miller et al. [50], who studied postmenopausal women without previous CVD in 36 U.S. metropolitan areas from 1994 to 1998. They estimated that each 10 µg/m^3^increase in PM_2.5_ was associated with a 24% increase in the risk of a cardiovascular event (hazard ratio, 1.24; 95% CI = 1.09 to 1.41). Those events would almost certainly have resulted in hospitalizations.

These findings also clarify a previous apparent inconsistency. Cohort studies of the association of PM2.5 and deaths from CVD or stroke have reported much larger effect sizes than the time series studies of PM2.5 and admissions from those causes [51], [52], [53], [54]. This seems implausible since many of those events result in hospitalizations. However, these chronic mortality estimates are also much larger than the time series estimates of the acute effects of recent PM2.5 exposure on deaths from those causes. The usual explanation is that chronic exposure produces greater effects because it leads to cumulative damage, such as atherosclerosis etc. [55], [56], [57], [58], [59]. Those arguments would be equally applicable to the effects of long term exposure on chronic rates of admissions for these causes. In this paper we show that such larger effects in fact are seen.

A major limitation of this study is our limited ability to control for individual level potential confounders, such as socio-economic factors, diet, exercise, etc. We have used area-based measures of socio-economic factors. To test the potential for confounding, we used data from the Normative Aging Study [60], [61], a population based study of an aging cohort, resident in Maine, Massachusetts, New Hampshire and Rhode Island As a general population of subjects eligible for Medicare, we think this is a reasonable test of the potential for confounding. We assigned the same 365 day average exposure to those participants from our model, and examined the association with packyears, with physical activity (METS), and with dietary fish intake. In no case was there a significant association.

Another limitation of the present study is the relatively coarse spatial resolution of 10×10 km. However, as satellite remote sensing evolves and progresses, higher spatial resolution data (3×3 km and 1×1 km) should become available which will further reduce exposure error. Such finer resolution should enable us to assess more precise estimated daily individual exposure as they relate to different location such as residence, work place etc.

In conclusion, we have demonstrated how our prediction models perform well in assessing short term and long term human exposures. Our findings indicate that hospital admission were associated with both short term and long term exposure to PM_2.5_. These findings present new opportunities to study the effects of both the long and short term exposure and human health.

## Supporting Information

Appendix S1 Crude estimates vs. Final model estimates.
(DOCX)

Appendix S2 Estimates of model covariates.
(DOCX)
