# Supplementary material for: Acute and Chronic Effects of Particles on Hospital Admissions in New-England
Source: PLoS One. 2012 Apr 17;7(4):e34664. doi: 10.1371/journal.pone.0034664 (PMC3328473; doi:10.1371/journal.pone.0034664)
Supplement: Appendix S1 — Crude estimates vs. Final model estimates. (DOCX) [file pone.0034664.s001.docx]

**Appendix S1- Crude estimates vs. Final model estimates**

| **Crude** |  |  |  |  |  |  |  |
| --- | --- | --- | --- | --- | --- | --- | --- |
|  |  |  |  |  |  |  |  |
| **CVD** | **Value** | **S.E** | **t** | **p** | **%** | **95% CI (lower/upper)** | |
| Acute PM | 0.001 | 0.000 | 6.471 | 0.000 | 1.024 | 0.713 | 0.467 |
| Chronic PM | 0.004 | 0.001 | 2.631 | 0.009 | 3.813 | 0.959 | 4.299 |
|  |  |  |  |  |  |  |  |
| **All respiratory** | **Value** | **S.E** | **t** | **p** | **%** | **95% CI (lower/upper)** | |
| Acute PM | 0.001 | 0.000 | 3.880 | 0.000 | 0.686 | 0.339 | 0.523 |
| Chronic PM | 0.005 | 0.002 | 3.217 | 0.001 | 5.197 | 2.000 | 4.772 |
|  |  |  |  |  |  |  |  |
| **Diabetes** | **Value** | **S.E** | **t** | **p** | **%** | **95% CI (lower/upper)** | |
| Acute PM | 0.001 | 0.000 | 5.485 | 0.000 | 0.946 | 0.607 | 0.510 |
| Chronic PM | 0.007 | 0.002 | 4.783 | 0.000 | 7.564 | 4.398 | 4.616 |
|  |  |  |  |  |  |  |  |
| **Storke** | **Value** | **S.E** | **t** | **p** | **%** | **95% CI (lower/upper)** | |
| Acute PM | 0.000 | 0.000 | 0.997 | 0.319 | 0.189 | -0.182 | 0.562 |
| Chronic PM | 0.007 | 0.002 | 3.810 | 0.000 | 6.864 | 3.276 | 5.293 |
|  |  |  |  |  |  |  |  |
| **Final** |  |  |  |  |  |  |  |
|  |  |  |  |  |  |  |  |
| **CVD** | **Value** | **S.E** | **t** | **p** | **%** | **95% CI (lower/upper)** | |
| Acute PM | 0.00103 | 0.000 | 6.514 | 0.000 | 1.03 | 0.72 | 0.47 |
| Chronic PM | 0.00308 | 0.001 | 2.169 | 0.030 | 3.12 | 0.30 | 4.29 |
|  |  |  |  |  |  |  |  |
| **All respiratory** | **Value** | **S.E** | **t** | **p** | **%** | **95% CI (lower/upper)** | |
| Acute PM | 0.001 | 0.000 | 3.937 | 0.000 | 0.70 | 0.35 | 0.52 |
| Chronic PM | 0.004 | 0.002 | 2.634 | 0.008 | 4.22 | 1.06 | 4.75 |
|  |  |  |  |  |  |  |  |
| **Diabetes** | **Value** | **S.E** | **t** | **p** | **%** | **95% CI (lower/upper)** | |
| Acute PM | 0.001 | 0.000 | 5.549 | 0.000 | 0.96 | 0.62 | 0.51 |
| Chronic PM | 0.006 | 0.002 | 4.047 | 0.000 | 6.33 | 3.22 | 4.59 |
|  |  |  |  |  |  |  |  |
| **Storke** | **Value** | **S.E** | **t** | **p** | **%** | **95% CI (lower/upper)** | |
| Acute PM | 0.000 | 0.000 | 1.268 | 0.205 | 0.24 | -0.13 | 0.56 |
| Chronic PM | 0.003 | 0.002 | 2.012 | 0.044 | 3.49 | 0.09 | 5.18 |
