# Supplementary material for: Acute and Chronic Effects of Particles on Hospital Admissions in New-England
Source: PLoS One. 2012 Apr 17;7(4):e34664. doi: 10.1371/journal.pone.0034664 (PMC3328473; doi:10.1371/journal.pone.0034664)
Supplement: Appendix S2 — Estimates of model covariates. (DOCX) [file pone.0034664.s002.docx]

**Appendix S2- Estimates of model covariates**

| **All respiratory** |  |  |  |  |
| --- | --- | --- | --- | --- |
|  | **B** | **S.E** | **t** | **p** |
| Acute PM | 0.001 | 0.000 | 3.937 | 0.000 |
| Chronic PM | 0.004 | 0.002 | 2.634 | 0.008 |
| Temperature | 0.002 | 0.000 | 13.842 | 0.000 |
| Median income | 0.028 | 0.000 | 5.524 | 0.000 |
| Average percent minorities | 0.017 | 0.001 | 16.798 | 0.000 |
| Average percent no high school degree | 0.007 | 0.001 | 4.921 | 0.000 |
| Average percent above 65 years | 0.008 | 0.002 | 4.533 | 0.000 |
| **CVD** |  |  |  |  |
|  | **B** | **S.E** | **t** | **p** |
| Acute PM | 0.001 | 0.000 | 6.514 | 0.000 |
| Chronic PM | 0.003 | 0.001 | 2.169 | 0.030 |
| Temperature | 0.001 | 0.000 | 5.790 | 0.000 |
| Median income | 0.035 | 0.000 | 5.950 | 0.000 |
| Average percent minorities | 0.021 | 0.001 | 18.229 | 0.000 |
| Average percent no high school degree | 0.008 | 0.002 | 4.805 | 0.000 |
| Average percent above 65 years | 0.010 | 0.002 | 5.020 | 0.000 |
| **Diabetes** |  |  |  |  |
|  | **B** | **S.E** | **t** | **p** |
| Acute PM | 0.001 | 0.000 | 5.549 | 0.000 |
| Chronic PM | 0.006 | 0.002 | 4.047 | 0.000 |
| Temperature | 0.001 | 0.000 | 7.378 | 0.000 |
| Median income | 0.019 | 0.000 | 4.073 | 0.000 |
| Average percent minorities | 0.018 | 0.001 | 19.369 | 0.000 |
| Average percent no high school degree | 0.006 | 0.001 | 4.594 | 0.000 |
| Average percent above 65 years | 0.005 | 0.002 | 3.292 | 0.001 |
| **Stroke** |  |  |  |  |
|  | **B** | **S.E** | **t** | **p** |
| Acute PM | 0.000 | 0.000 | 1.268 | 0.205 |
| Chronic PM | 0.003 | 0.002 | 2.012 | 0.044 |
| Temperature | 0.000 | 0.000 | -0.914 | 0.361 |
| Median income | 0.007 | 0.000 | 3.567 | 0.000 |
| Average percent minorities | 0.005 | 0.000 | 15.043 | 0.000 |
| Average percent no high school degree | 0.001 | 0.001 | 2.567 | 0.010 |
| Average percent above 65 years | 0.002 | 0.001 | 3.680 | 0.000 |
